# Editorial: Antimicrobial secondary metabolites: sources and opportunities to devise biocontrol strategies to counteract phytopathogens

**DOI:** 10.3389/fpls.2024.1382604

**Published:** 2024-02-23

**Authors:** Maria Julissa Ek-Ramos, Saúl Fraire-Velázquez

**Affiliations:** ^1^ Department of Microbiology and Immunology, School of Biological Sciences, Autonomous University of Nuevo Leon, San Nicolás de los Garza, Mexico; ^2^ Lab. Integrative Biology of Plants and Microorganisms, Autonomous University of Zacatecas, Academic Unit of Biological Science, Zacatecas, Mexico

**Keywords:** antimicrobial, phytopathogens, biocontrol, secondary metabolites, natural antimicrobials

On a global scale, the excessive use of pesticides in agriculture reaches nearly 3 million tons annually. However, their management is commonly characterized by very low efficiency, as seen in the case of insecticides, which barely achieve a 1% effectiveness rate (Tudi et al., 2021). This practice therefore results in high contamination of soil and water natural resources and adversely impacts the biodiversity of macro and microorganisms. Additionally, it leads to issues of worker intoxication in the fields. Considering the social pressure from consumers who are increasingly aware and demand safe agri-food products free of agrochemicals, we are compelled to combat phytopathogens through research looking for new alternatives. The use of secondary metabolites presents great potential; good examples of sources are microorganisms such as fungi and bacteria, which produce antibiotics, and a broad diversity of plants that also offers many opportunities in this regard. This Research Topic in Frontiers in Plant Science presents recent reports that show the importance and effects of novel secondary metabolites with potential to be used against phytopathogens. The increasing number of microorganisms which sequenced genomes are available, particularly those capable of promote plant growth and/or inhibit pathogens, supported by metabolomics studies alongside with genome mining, opens up enormous possibilities for expanding the inventory of beneficial microorganisms strains and biochemical compounds with antimicrobial properties. Extraction cocktails of individual or consortia of microorganisms can be developed, with the objective to contain secondary metabolites with high specificities towards recalcitrant phytopathogens in crops of global or regional socioeconomic importance. In this scenario, the focus is on exploring options for implementing anti-phytopathogenic metabolites from bacteria, fungi, and plants. As concrete examples of useful microorganisms, Jeon et al., reported the identification of a peptide produced by a bacterium of the genus *Streptomyces*, which has been proven to exhibit antimicrobial activity against phytopathogenic bacteria and fungi. Most importantly, the synthesized and purified peptide has been used to control *Pseudomonas syringae* pv. tomato DC3000 infection in *Arabidopsis thaliana* plants. Furthermore, the respective gene for this metabolite is highly conserved in the genus *Streptomyces*. Similarly, Ghareeb et al., has been reported that aqueous and methanolic extracts from the bacterium *Oscillatoria* sp. demonstrate antinematicidal activity against *Meloidogyne incognita*, a parasite that greatly impacts soybean cultivation. Additionally, Santra and Banerjee, have shown that an endophytic strain of the fungus *Diaporthe* sp. and a strain of *Curvularia* sp. exhibit antifungal activity through volatile and non-volatile metabolites which inhibit eight phytopathogenic fungi, leading to a lethal loss of proteins and other cellular components in the pathogen. On the other hand, expanding our knowledge about the virulence factors shared by several bacterial phytopathogens, which they utilize to colonize and infect diverse hosts, is highly beneficial. This knowledge allows for conducting *in vitro* and *in silico* interactome studies to search for secondary metabolites, cellular components, or simpler specific molecules from various sources that can inhibit the biological function of the pathogen´s virulence component. A great example of these new findings is reported by Park et al., where it has been shown that bacteria such as *Xanthomonas oryzae* pv. *oryzae*, *Ralstonia solanacearum* and *Burkholderia glumae* share virulence factors such as C4-dicarboxylate ABC transporter, flagellar biosynthesis protein, protocatechuate 3,4-dioxygenase (PCD) and 2-methylisocitrate lyase. Notably, the corresponding gene for PCD is clearly involved in virulence, as evidenced by gene disruption tests conducted in all three pathogens. For concrete examples of metabolites from plants, Hari et al., have been shown that *Origanum elongatum*, a medicinal plant found in Morocco, contains various compounds with important biological activities. It has been reported that 56 components are in the most active extract that include phenolic acids, flavonoids, organic acids, tannins, and coumarins, attributing the antifungal effects to compounds like syringic acid and rosmarinic acid. Most importantly it has been found that these extracts have impact on late blight disease severity, with promising results suggesting the potential use of *O. elongatum* extracts as eco-friendly alternatives for plant disease management of *Phytophtora infestans*. Furthermore, Nadeem et al., have been reported that other interesting plant, *Nepeta cataria*, historically used for various medicinal purposes due to its analgesic, anti-asthmatic, anti-cancer, anti-inflammatory and antimicrobial properties, contains essential oils, flavonoids, phenolic acids, steroids, terpenoids, and terpenoid hydrocarbons. Studies highlight its potential in treating diarrhea, its impact on industrial food materials, its inhibitory effects on herpes virus replication, its use during the COVID-19 pandemic and its effectiveness against *Aedes aegypti* for dengue prevention.

These new findings have the potential to be developed into new products, such as Curaderm®, which is recommended to treat several diseases and has been approved by the FDA as a medicinal entity. It contains a mixture of secondary metabolites recognized as natural product botanicals (Chase et al., 2020; Gallardo and Seca, 2022). Another example is the formulation of biopesticides, such as those containing metabolites produced by *Chromobacterium subtsugae*, combined with pigments and proteins possessing repellent and antifeeding properties. These are sold as the insecticide Grandevo® (Marrone, 2019; Gallardo and Seca, 2022). In conclusion, research efforts like the one presented in this Research Topic, aimed at isolating novel secondary metabolites from diverse natural sources and developing new methodologies for their extraction, identification and testing, should be supported to optimize their future applications.

## Author contributions

ME-R: Writing – original draft, Writing – review & editing. SF-V: Writing – original draft, Writing – review & editing.
